# Effects of thyroid hormone on mitochondria and metabolism of human preimplantation embryos

**DOI:** 10.1002/stem.3129

**Published:** 2019-12-26

**Authors:** Laila Noli, Shirin E. Khorsandi, Angela Pyle, Gnanaratnam Giritharan, Norah Fogarty, Antonio Capalbo, Liani Devito, Vladimir M. Jovanovic, Preeti Khurana, Hannah Rosa, Nikola Kolundzic, Aleksandra Cvoro, Kathy K. Niakan, Afshan Malik, Russell Foulk, Nigel Heaton, Mohammad Saleh Ardawi, Patrick F. Chinnery, Caroline Ogilvie, Yacoub Khalaf, Dusko Ilic

**Affiliations:** ^1^ Division of Women's and Children's Health, Faculty of Life Sciences and Medicine King's College London and Assisted Conception Unit, Guy's Hospital London UK; ^2^ Department of Pathological Sciences Fakeeh College for Medical Sciences Jeddah Saudi Arabia; ^3^ Institute of Liver Studies, King's College Hospital London UK; ^4^ Wellcome Trust Centre for Mitochondrial Research Institute of Genetic Medicine, Newcastle University Newcastle upon Tyne UK; ^5^ Nevada Center for Reproductive Medicine Reno Nevada; ^6^ Human Embryo and Stem Cell Laboratory The Francis Crick Institute London UK; ^7^ Igenomix Italy via Fermi 1, Marostica Italy; ^8^ DAHFMO, Unit of Histology and Medical Embryology, Sapienza, University of Rome Rome Italy; ^9^ Bioinformatics Solution Center and Human Biology Group; Institute for Zoology; Department of Biology, Chemistry and Pharmacy Freie Universität Berlin Berlin Germany; ^10^ MitoDNA Service Lab King's College London London UK; ^11^ Center for Bioenergetics Houston Methodist Research Institute Houston Texas; ^12^ MRC‐Mitochondrial Biology Unit and Department of Clinical Neurosciences University of Cambridge Cambridge UK; ^13^ Department of Medical and Molecular Genetics King's College London London UK

**Keywords:** embryo development, mitochondria, oxidative phosphorylation, T3, thyroid hormone

## Abstract

Thyroid hormones are regarded as the major controllers of metabolic rate and oxygen consumption in mammals. Although it has been demonstrated that thyroid hormone supplementation improves bovine embryo development in vitro, the cellular mechanisms underlying these effects are so far unknown. In this study, we investigated the role of thyroid hormone in development of human preimplantation embryos. Embryos were cultured in the presence or absence of 10^−7^ M triiodothyronine (T3) till blastocyst stage. Inner cell mass (ICM) and trophectoderm (TE) were separated mechanically and subjected to RNAseq or quantification of mitochondrial DNA copy number. Analyses were performed using DESeq (v1.16.0 on R v3.1.3), MeV4.9 and MitoMiner 4.0^v2018 JUN^ platforms. We found that the exposure of human preimplantation embryos to T3 had a profound impact on nuclear gene transcription only in the cells of ICM (1178 regulated genes—10.5% of 11 196 expressed genes) and almost no effect on cells of TE (38 regulated genes—0.3% of expressed genes). The analyses suggest that T3 induces in ICM a shift in ribosome and oxidative phosphorylation activity, as the upregulated genes are contributing to the composition and organization of the respiratory chain and associated cofactors involved in mitoribosome assembly and stability. Furthermore, a number of genes affecting the citric acid cycle energy production have reduced expression. Our findings might explain why thyroid disorders in women have been associated with reduced fertility and adverse pregnancy outcome. Our data also raise a possibility that supplementation of culture media with T3 may improve outcomes for women undergoing in vitro fertilization.

## Graphical abstract

Embryos cultured in the presence or absence of triiodothyronine (T3) till blastocyst stage upregulate genes contributing to the composition and organization of the respiratory chain and associated cofactors involved in mitoribosome assembly and stability. Furthermore, a number of genes affecting the citric acid cycle energy production have reduced expression.

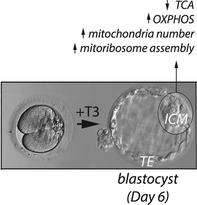

**Significance statement**

Thyroid hormones are regarded as the major controllers of metabolic rate and oxygen consumption in mammals. Little is known about the effects of the thyroid hormones in the earliest stages of human development. The results suggest that thyroid hormones affect mitochondrial function in human embryos: stimulate mitochondrial replication and energy production within mitochondria by switching metabolism from glycolytic pathway to more efficient oxidative phosphorylation. The findings shed a light on metabolic switch in early embryo development and might explain why thyroid disorders in women have been associated with reduced fertility and adverse pregnancy outcome. Data in the present study also suggest that supplementation of culture media with T3 may improve outcomes for women undergoing in vitro fertilization.

## 1 INTRODUCTION

Thyroid hormones, thyroxine (T4), and its biologically active form triiodothyronine (T3) play vital roles in regulating homeostasis and metabolic rate of human cells and tissues. They are essential for physical and mental development; insufficient production of thyroid hormones before birth or during childhood can lead to reduced growth and mental impairment. In adults, hypothyroidism causes reduced metabolism, poor memory, depression, and reduced fertility.

In women of fertile age, hypothyroidism frequently results in menstrual irregularities; ovulation and conception are possible, though resulting pregnancies are liable to miscarry. In addition, women with severe hypothyroidism have been reported to have diminished libido and ovulation failure.^1^

Thyroid hormone is detected in follicular fluid and has a role in ovarian follicle cycle and egg maturation.^2^ Evidence is now emerging on the importance of thyroid hormone signaling during implantation.^3^ Thyroid hormone receptors α (*THRA*) and β (*THRB*) transcripts and protein levels were detected in developing bovine preimplantation embryos up to the blastocyst stage.^4^ The exposure of preimplantation embryos to T3 in culture medium had a positive effect on their development—a greater viability, higher hatching rate, significant increase in total cell number of blastocysts, as well as a significantly lower number of apoptotic cells.^5^, ^6^

Exposure to T3 of human embryonic stem cells (hESCs) derived from the inner cell mass (ICM) of the blastocyst stage embryos and induced pluripotent stem cells triggered expression of multiple genes linked to regulation of gene transcription, cell cycle, morphology, apoptosis, cell viability, and cellular and embryonic development.^7^

Here, we report investigations into the effect of thyroid hormone on the transcriptome of developing human preimplantation embryos and show that ICM and trophectoderm (TE) react differently to T3 exposure. Whereas T3 had a profound effect on gene expression in the ICM, TE cells remained almost unaffected.

## 2 MATERIALS AND METHODS

### 2.1 Materials

The work described here was done under license from the UK Human Fertilisation and Embryology Authority (research license number: R0075) as well as local ethical approvals (the Institutional Review Board for the embryos from the Nevada Center for Reproductive Medicine and UK National Health Service Research Ethics Committee Reference: 06/Q0702/90 for the embryos from the United Kingdom). Informed consent was obtained from all subjects, and the experiments conformed to the principles set out in the WMA Declaration of Helsinki and the NIH Belmont Report. No financial inducements were offered for donation.

### 2.2 Human embryo culture

The embryos were thawed using Quinn's Advantage thaw kit according to the manufacturer's instructions (Sage). After thawing, the embryos were cultured in 40‐μL microdrops of Quinn's medium supplemented with 10% synthetic protein serum substitute (Sage) under mineral oil (Sage) at 37°C in 5% CO_2_ and 5% O_2_ in a humidified atmosphere. Treated embryos were exposed from thawing till harvesting to 10^−7^ M T3 (Sigma). The medium was refreshed on a daily basis. ICM and TE were separated mechanically as described.^8^, ^9^

### 2.3 RNAseq and data analysis

The recovered ICM and TE fractions were stored at −80°C till processing. cDNA was prepared using SMARTer Ultra Low Input RNA for Illumina Sequencing—HV (Clontech) directly from cells. All experiments were carried out in a PCR clean workstation using protocols provided by the manufacturer. The purification of the first‐strand cDNA was carried out using SPRI Ampure Beads (Beckman Coulter). The amplification of the ds cDNA was carried out in a PCR Clean workstation using an Advantage 2 PCR Kit as per the manufacturer's protocol. The PCR‐amplified cDNA was purified using immobilization onto SPRI beads. One microliter of the amplified cDNA was used for the validation using the Agilent 2100 BioAnalyzer and the High Sensitivity DNA Chip (Agilent) per the manufacturer's instructions. The Covaris AFA system was used for the controlled shearing of DNA prior to the generation of the library for Illumina sequencing. The shearing system led to the formation of DNA sequences in the range of 200‐500 bp. Ten microliters of the fragmented ds‐cDNA sample was used for library preparation. The library purification protocol was similar to that used to purify cDNA. The libraries were sequenced on HiSeq 2000 system (Illumina). The libraries were quantified using Qubit (Life Technologies), and the size profile was analyzed on the 2200 TapeStation (Agilent).

GenoSplice Technology performed quality control (QC), processing, and further analyses of the data. For each gene present in FAST DB v2014_2, reads aligning on constitutive regions (that are not prone to alternative splicing) were counted. Based on these read counts, normalization and differential gene expression were performed using DESeq (v1.16.0 on R v3.1.3).^10^ Sample clustering was performed with MeV4.9^11^ using correlation of normalized read counts of expressed genes and average linkage clustering.

### 2.4 Mitochondrial gene network analysis

Genes identified to have significant differential expression (n = 1178; fold change ≥1.5, *P* ≤ .05; GenoSplice, EASANA) were filtered for mitochondrial identity in MitoMiner 4.0^v2018 JUN^ (accessed December 2018) as defined by the integrated mitochondrial protein index as “KNOWN” mitochondrial.^12^ Uniprot identifier of “KNOWN” mitochondrial proteins were imported into STRING version 10.5 (last accessed December 2018), and a k‐means network was built using an interaction score set at a confidence of 0.7 using the whole human genome as background statistical reference.^13^

### 2.5 Quantification of mtDNA copy number

Quantification of mtDNA copy number was performed as described.^14^ Briefly, quantitative real‐time PCR (qPCR) was performed on a CFX96 Touch Real‐Time PCR Detection System (Bio‐Rad). mtDNA copy number was calculated by absolute quantification using a singleplex Taqman assay targeting the mitochondrial MT‐ND1 gene.

## 3 RESULTS

### 3.1 T3 modifies gene expression only in ICM and not in TE

**Figure 1:**
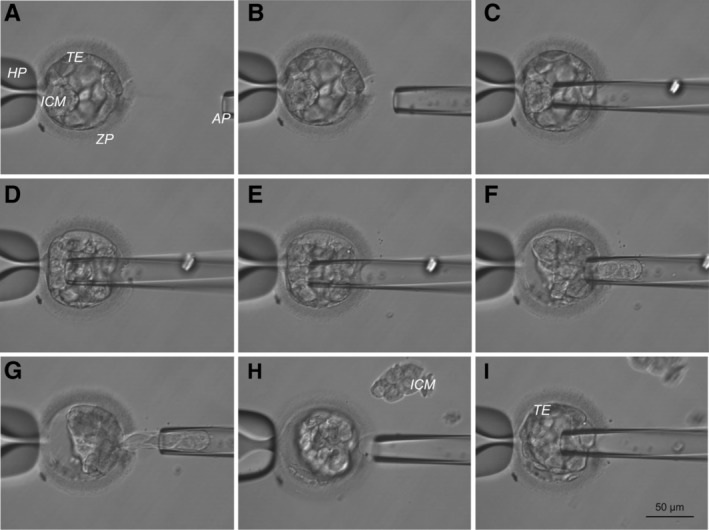
Biopsy of inner cell mass (ICM). A‐I, Images illustrate mechanical separation of ICM from trophectoderm (TE). AP, aspiration pipette; HP, holding pipette; ZP, zona pellucida

**Figure 2:**
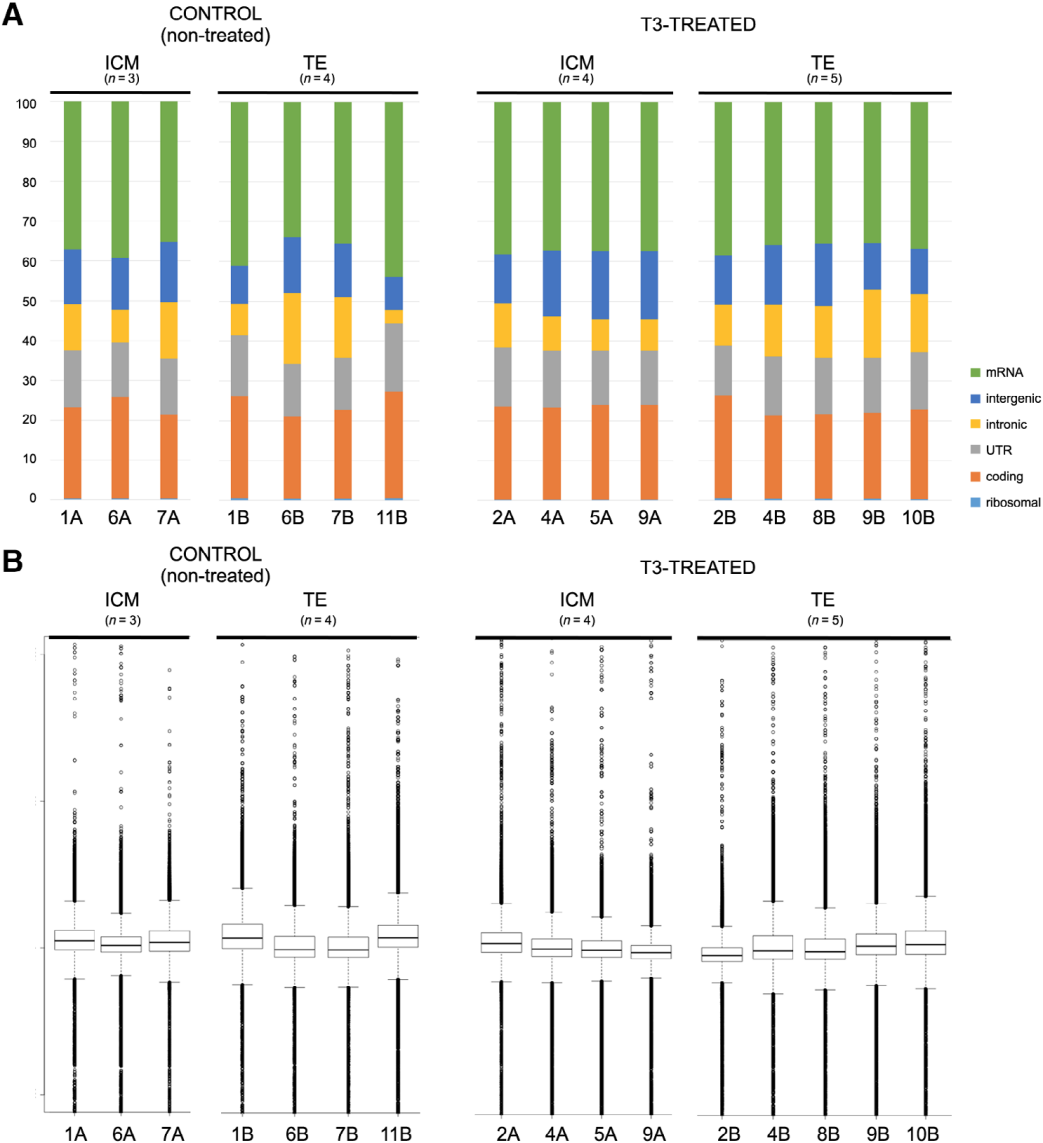
Quality control of RNAseq data. A, Mapped read annotations. Read annotations are homogeneous distributed and have a high proportion of “usable reads” for all samples. B, Estimation of insert size. Mapping statistics shows medium/high proportion of mapped reads (cca 84%). No or few overlap between forward and reverse reads. No bias regarding 5′3′ coverage of transcripts has been detected

A total of 30 embryos at the two pronuclear (2PN) stage were used for the experiment; 15 embryos were exposed to 10^−7^ M T3 and the remaining 15 embryos acted as controls. Six treated embryos and four control embryos developed into grade 1 blastocysts by day 6. The ICM was separated from TE for each of the 10 blastocysts (Figure 1), and RNAseq was used to evaluate the transcriptome of three ICM and four TE samples from control embryos and four ICM and five TE samples from treated embryos (Figure 2). The remaining ICM and TE samples were either mechanically damaged during handling or did not pass tiered QC of cDNA and library preparation.

**Figure 3:**
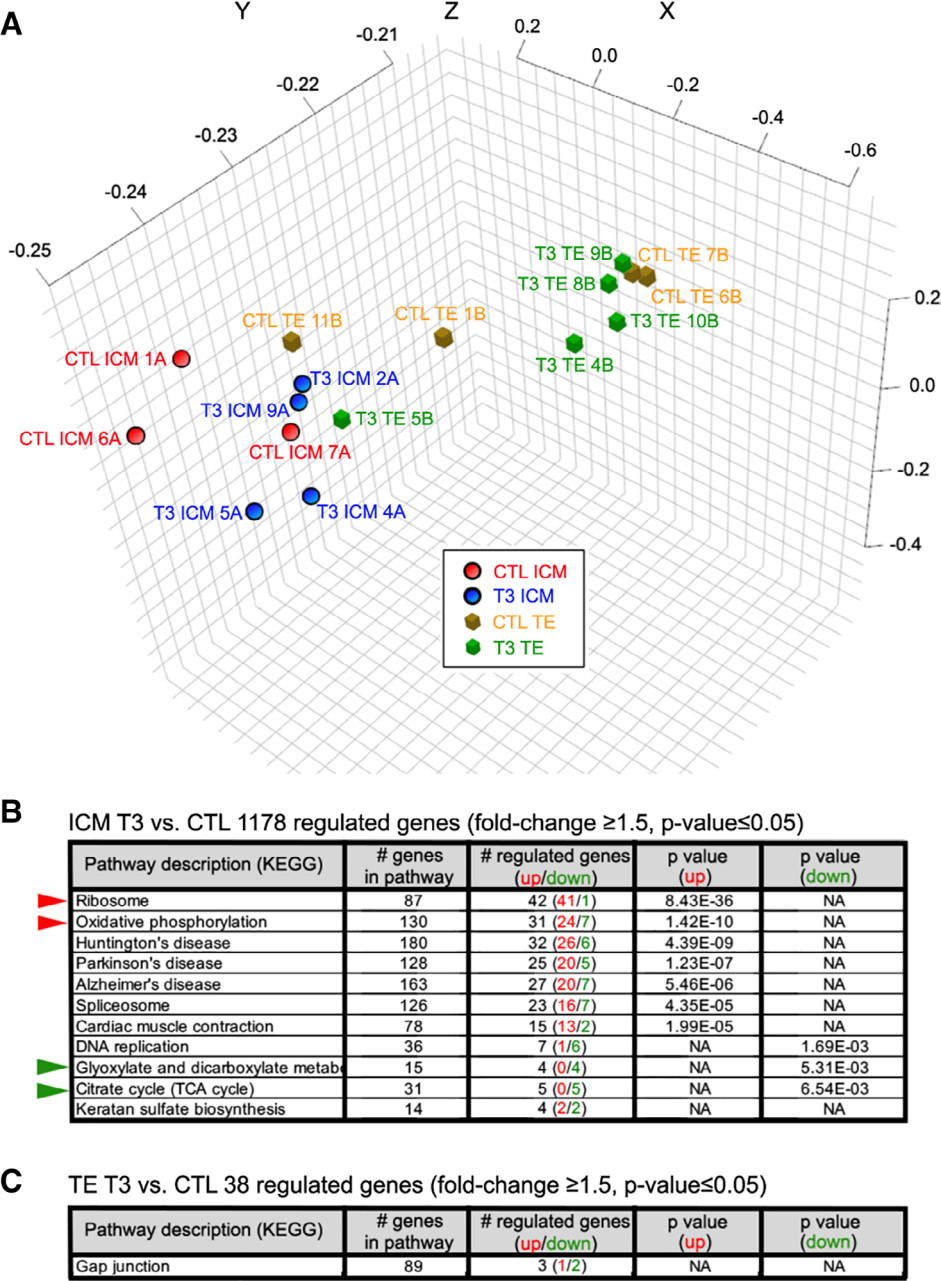
Effects of T3 exposure on transcriptome of human preimplantation embryos. Human 2pn embryos were cultured to the blastocyst stage either in the standard culture medium or in the medium supplemented with 100 nM T3. Inner cell mass was separated mechanically from trophectoderm (TE) (Figure 1), and the transcriptome has been analyzed with RNAseq using Illumina's HiSeq platform (Figure 2). A, Principal component analysis of samples: untreated control (CTL) ICM (n = 3) and TE (n = 4) and T3‐treated ICM (n = 4) and TE (n = 6). Cumulative proportion of variance: 82.4%. B, Analysis of differential gene expression between ICM of embryos cultured in a standard medium and in the medium supplemented with 100 nM T3 has identified 1178 genes (fold change ≥1.5, *P* ≤ .05). Kyoto Encyclopedia of Genes and Genomes database singled out 11 pathways regulated by T3. The most significantly upregulated are genes in *Ribosome* and *Oxidative phosphorylation* pathways (red arrow heads) and downregulated genes in *Glyoxylate and dicarboxylate metabolism* and *Citrate* (TCA) cycle (green arrowheads). Taken together, the data suggest a metabolic switch from glycolysis to oxidative phosphorylation accompanied with an increase in protein synthesis (*Ribosome* pathway). C, Analysis of differential gene expression between TE of embryos cultured in a standard medium and in the medium supplemented with 100 nM T3 has identified only 38 genes (fold change ≥1.5, *P* ≤ .05) suggesting that TE cells do not respond to thyroid hormone in the same way as the cells from ICM

Lineage specific markers for ICM (*NANOG*, *NODAL*, *SOX2*) and TE (*CDX2*, *GATA2*, *GATA3*) demonstrated separation of two subpopulations (Figure S1) The transcriptome analysis showed that low levels of both thyroid hormone receptors, *THRA* and *THRB*, are expressed in human preimplantation embryos, though predominantly in ICM (Figure S2). A total of 11 196 genes were considered as expressed in the ICM and 12 238 genes in the TE and analyzed for differential expression. There were 1178 differently regulated genes (10.5% of expressed genes) between ICM of control and ICM of T3‐exposed embryos (fold change ≥1.5 and *P*‐value ≤.05), whereas the difference between TE of control and TE of T3‐treated embryos was only in 38 genes (0.3% of expressed genes), indicating that the effect of T3 on ICM was profoundly different from its effect on TE (Figure 3; Table S1). Kyoto Encyclopedia of Genes and Genomes (KEGG) database singled out 11 pathways affected by T3 in ICM. The most significantly upregulated were genes in *Ribosome* and *Oxidative phosphorylation* (OXPHOS) pathways, and those downregulated were genes in *Glyoxylate and dicarboxylate metabolism* and *Citrate (TCA)* cycle (Figure 3B, C). Data suggest that T3 promoted a metabolic switch from glycolysis to OXPHOS accompanied with an increase in protein synthesis.

### 3.2 Mitochondrial bioenergetic differential events in silico

**Figure 4:**
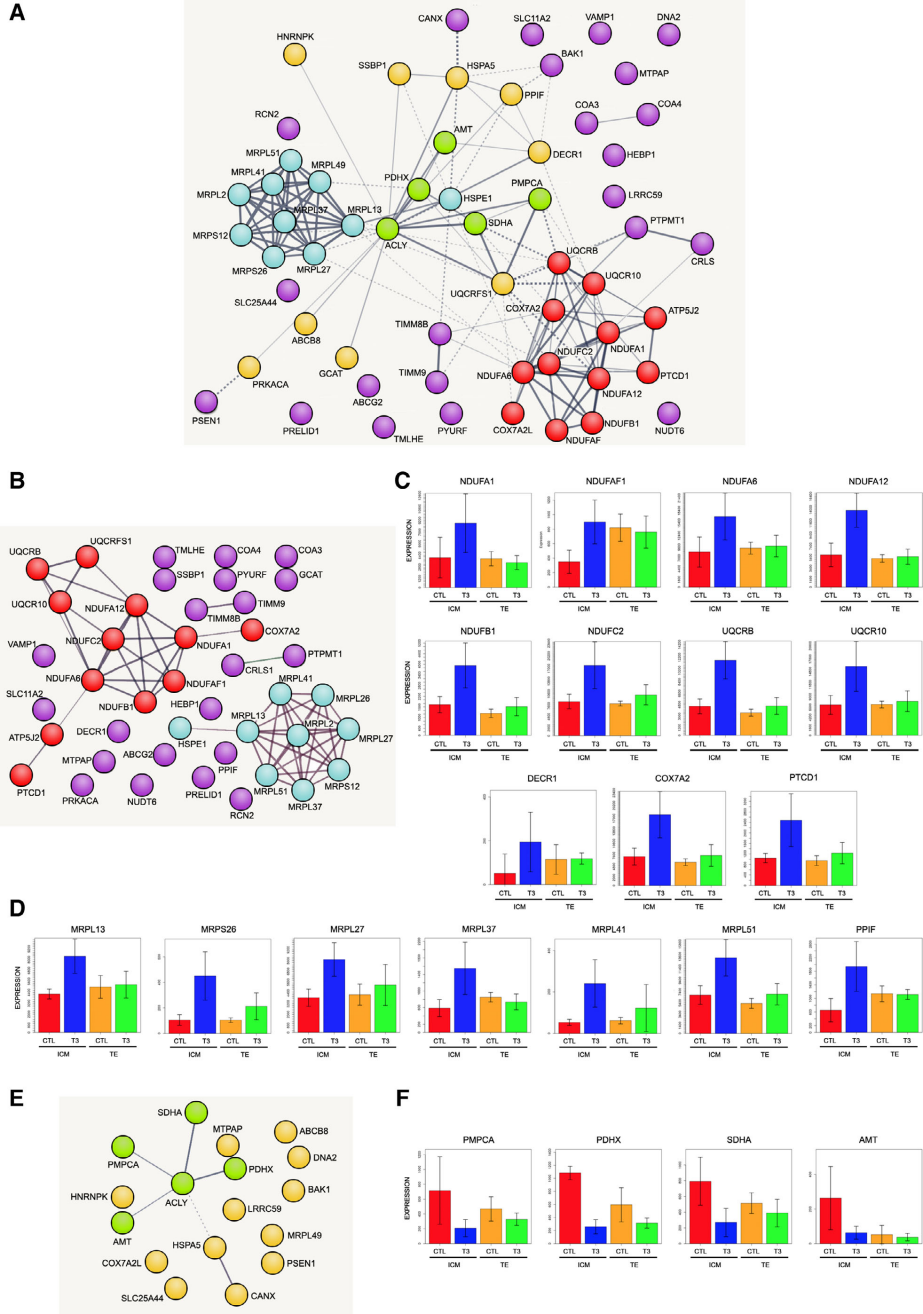
In silico analysis of mitochondrial bioenergetic differential events. A, Network of mitochondrial genes changed on T3 exposure. The two dominant clusters of the k‐means network are composed of protein subunits involved in respiratory chain architecture (red circles) and the mitoribosome (blue circles). Upregulated mitochondrial genes that are not directly part of either respiratory chain or mitoribosome are in purple. Green and orange circles represent mitochondrial genes that are significantly downregulated. B, Network of upregulated mitochondrial genes suggest shift in ribosome and oxidative phosphorylation activity. Red circles, respiratory chain proteins; blue circles, mitoribosome; purple circles, other upregulated mitochondrial genes. C, Changes of individual genes involved in respiratory chain architecture (correspond to red circles in A and B). D, Changes of individual genes involved in mitoribosome architecture (correspond to blue circles in A and B). E, Network of downregulated mitochondrial genes is primarily limited to the tricarboxylic acid cycle (TCA; green circles). Orange circles, other downregulated mitochondrial genes. F, Changes of individual genes affecting TCA cycle energy production (correspond to green circles in A and E)

In an attempt to obtain an integrated picture of T3 effect on energy metabolism in ICM, we filtered the 1178 genes identified to have significant differential expression for mitochondrial identity. Among these genes, 42 mitochondrial genes were upregulated and 17 downregulated (Table 1). The k‐means built network based on these mitochondrial proteins is illustrated in Figure 4A. The top functional enrichments for the network are the respiratory electron transport chain (GO:0022904, FDR 7.66e‐12, n = 12 genes) and for mitochondrial organization (GO:0007005, FDR 3.12e‐11, n = 16 genes). This change in the ICM mitochondrial proteome on T3 exposure is demonstrated by the two dominant clusters of the k‐means network that are composed of protein subunits involved in respiratory chain architecture and the mitoribosome (Figure 4A). Other mitochondrial events identified in silico are related to mitochondrial membrane interactions and the intrinsic apoptotic pathway, while changes in the import and export of iron and calcium also appear to be affected (Table 1).

**Table 1:** List of 42 upregulated and 17 downregulated mitochondrial genes following exposure to T3

Gene protein name, Uniprot accession number	Mitochondrial function
**UP**	
HEBP1, Heme binding protein 1, Q9NRV9	Orphan protein inner mitochondrial space (IMS) calcium mobilization and chemotaxis
DECR1, 2,4‐dienoyl CoA reductase 1, Q16698	Auxiliary enzyme of beta‐oxidation
PPIF, Peptidylprolyl isomerase F, P30405	Involved in regulation of the mitochondrial permeability transition pore
MRPL27, Mitochondrial ribosomal protein L27, Q9P0M9	Mitoribosome subunit involved in translating mitochondrial mRNA
MRPL51, Mitochondrial ribosomal protein L51, Q4U2R6	Mitoribosome subunit involved in translating mitochondrial mRNA
HSPE1, Heat shock 10 kDa protein 1, P61604	Mitochondrial protein biogenesis
ABCG2, ATP‐binding cassette, sub‐family G (WHITE), member 2, Q9UNQ0	Heme export from mitochondria
NDUFAF1, NADH dehydrogenase (ubiquinone) complex I, assembly factor 1, Q9Y375	Complex 1 assembly factor
MTPAP, Mitochondrial poly(A) polymerase, Q9NVV4	Stabilization of mitochondrial mRNA
SSBP1, Single‐stranded DNA binding protein 1, Q04837	Mitochondrial DNA replication
PYURF, PIGY upstream reading frame, Q96I23	Complex 1 activity regulator
NDUFC2, NADH dehydrogenase (ubiquinone) 1, subcomplex unknown, 2, O95298	Complex 1 subunit
UQCRB, Ubiquinol‐cytochrome c reductase binding protein, P14927	Complex 3 subunit
ATP5J2, ATP synthase, H+ transporting, mitochondrial Fo complex, subunit F2, P56134	Complex 5 (aka ATP Synthase) subunit
PRELID1, PRELI domain containing 1, Q9Y255	Negative regulator of mitochondrial apoptotic process
NUDT6, Nudix (nucleoside diphosphate linked moiety X)‐type motif 6, P53370	Role in cell proliferation and cell survival
UQCRFS1, Ubiquinol‐cytochrome c reductase, P47985	Role in Complex 3 biogenesis
MRPL13, Mitochondrial ribosomal protein L13, Q9BYD1	Mitoribosome subunit involved in translating mitochondrial mRNA
MRPS12, Mitochondrial ribosomal protein S12, O15235	Mitoribosome subunit involved in translating mitochondrial mRNA
PRKACA, Protein kinase, cAMP‐dependent, P17612	Negative regulator of Hedgehog (Hh) signaling pathway
PTPMT1, Protein tyrosine phosphatase, mitochondrial 1, Q8WUK0	Negative regulator of mitochondrial apoptotic process
NDUFA12, NADH dehydrogenase (ubiquinone) 1 alpha subcomplex, 12, Q9UI09	Complex 1 subunit
NDUFB1, NADH dehydrogenase (ubiquinone) 1 beta subcomplex, 1, O75438	Complex 1 subunit
UQCR10, Ubiquinol‐cytochrome c reductase, complex III subunit X, Q9UDW1	Complex 3 subunit
TMLHE, Trimethyllysine hydroxylase, epsilon, Q9NVH6	Carnitine biosynthesis and succinate generation
COA4, Cytochrome c oxidase assembly factor 4 homolog, Q9NYJ1	Complex 4 assembly factor
MRPL37, Mitochondrial ribosomal protein L37, Q9BZE1	Mitoribosome subunit involved in translating mitochondrial mRNA
COA3, Cytochrome c oxidase assembly factor 3, Q9Y2R0	Component of MITRAC (mitochondrial translation regulation assembly intermediate of cytochrome c oxidase complex) complex. Required for efficient translation of MT‐CO1 and mitochondrial respiratory chain Complex 4 assembly
COX7A2, Cytochrome c oxidase subunit VIIa polypeptide 2, P14406	Complex 4 subunit
NDUFA1, NADH dehydrogenase (ubiquinone) 1 alpha subcomplex, 1, O15239	Complex 1 subunit
MRPL41, Mitochondrial ribosomal protein L41, Q8IXM3	Mitoribosome subunit involved in translating mitochondrial mRNA
CRLS1, Cardiolipin synthase 1, Q9UJA2	Mitochondrial membrane integrity and dynamics
MRPS26, Mitochondrial ribosomal protein S26, Q9BYN8	Mitoribosome subunit involved in translating mitochondrial mRNA
GCAT, Glycine C‐acetyltransferase, O75600	Glycine synthesis, provides one‐carbon units to support the biosynthesis of nucleotides and amino acid
MRPL2, Mitochondrial ribosomal protein L2, Q5T653	Mitoribosome subunit involved in translating mitochondrial mRNA
RCN2, Reticulocalbin 2, EF‐hand calcium binding domain, Q14257	Orphan protein IMS involved in calcium binding
SLC11A2, Solute carrier family 11, P49281	Mitochondrial Iron import
TIMM9, Translocase of inner mitochondrial membrane 9, Q9Y5J7	Mitochondrial biogenesis
VAMP1, Vesicle‐associated membrane protein 1, P23763	Targeting of transport vesicles to mitochondria
NDUFA6, NADH dehydrogenase (ubiquinone) 1 alpha subcomplex, 6, P56556	Complex 1 subunit
TIMM8B, Translocase of inner mitochondrial membrane 8 homolog B, Q9Y5J9	Mitochondrial biogenesis
PTCD1, Pentatricopeptide repeat domain 1, O75127	Mitoribosome assembly and stability
**DOWN**	
LRRC59, Leucine rich repeat containing 59, Q96AG4	Mitochondrial fission via Dynamin Related Protein 1 (DRP1) interaction and modulates mitochondrial polymerase activity
PDHX, Pyruvate dehydrogenase complex, component X, O00330	Converts pyruvate to acetyl coenzyme A, links glycolysis to tricarboxylic acid cycle (TCA)
COX7A2L, Cytochrome c oxidase subunit VIIa polypeptide 2 like, O14548	Complex 3 cofactor influencing supercomplex formation
CANX, Calnexin, P27824	Interacts with mitochondrial associated membrane to alter mitochondrial fission
ACLY, ATP citrate lyase, P53396	Converts mitochondria‐derived citrate into oxaloacetate and acetyl‐CoA link between glycolysis and TCA cycle
MTPAP, Mitochondrial poly(A) polymerase, Q9NVV4	Polymerase that creates the 3′ poly(A) tail of mitochondrial transcripts.
SDHA, Succinate dehydrogenase complex, subunit A, P31040	Complex 2 subunit
AMT, Aminomethyltransferase, P48728	Degrades glycine
MRPL49, Mitochondrial ribosomal protein L49, Q13405	Mitoribosome subunit involved in translating mitochondrial mRNA
HSPA5, Heat shock 70 kDa protein 5, P11021	Bridge between endoplasmic reticulum for stress induced apoptosis
PSEN1, Presenilin 1, P49768	Involved in lipid metabolism and calcium homeostasis
ABCB8, ATP‐binding cassette, sub‐family B (MDR/TAP), member 8, Q9NUT2	Transport of heme and peptides, from mitochondria to the nucleus and cytosol. Transport of phospholipids into mitochondrial membranes
SLC25A44, Solute carrier family 25, member 44, Q96H78	Mitochondrial transporter, calcium‐dependent mitochondrial aspartate and glutamate carrier
BAK1, BCL2‐antagonist/killer 1, Q16611	Localizes to mitochondria proapoptotic
PMPCA, Peptidase (mitochondrial processing) alpha, Q10713	Cleaves presequences (transit peptides) from mitochondrial protein precursors and release of N‐terminal transit peptides from precursor proteins imported into mitochondria
HNRNPK, Heterogeneous nuclear ribonucleoprotein K, P61978	Anti‐apoptotic
DNA2, DNA replication helicase 2 homolog, P51530	Mitochondrial nuclease/helicase

To understand the change in mitochondrial bioenergetic profile further, we have divided k‐means network into an UP (Figure 4B‐D) and a DOWN (Figure 4E, F). A shift in OXPHOS activity appears to be occurring on T3 exposure, as all the mitochondrial related proteins identified in silico to have UP expression are subunits contributing to the composition and organization of Complexes 1, 3, 4, and 5 of the respiratory chain (Table 1, Figure 4B‐D). In parallel, there is a change in large and small subunit composition of the mitoribosome and associated cofactors involved in mitoribosome assembly and stability that would potentially alter translation of respiratory chain subunits encoded in the mitochondrial genome. DOWN expression of mitochondrial proteins is primarily limited to the tricarboxylic acid cycle (TCA), as demonstrated by the change in Succinate Dehydrogenase complex, subunit A which is both a catalytic subunit of Complex 2 of the respiratory chain and the TCA cycle where it converts succinate to fumarate. In association with this DOWN change, a number of enzymes involved in AcetylCoA generation which affects TCA cycle energy production have reduced expression (Table 1, Figure 4E, F).

### 3.3 T3 exposure is associated with higher levels of mtDNA in ICM

Since mitochondrial respiratory chain subunits are encoded by both nuclear and mitochondrial genomes, communication between nuclear and mtDNA is essential for mitochondrial function. To determine whether the replication of these genomes is coordinated following exposure to T3, we analyzed expression levels of mtDNA encoded genes.

**Figure 5:**
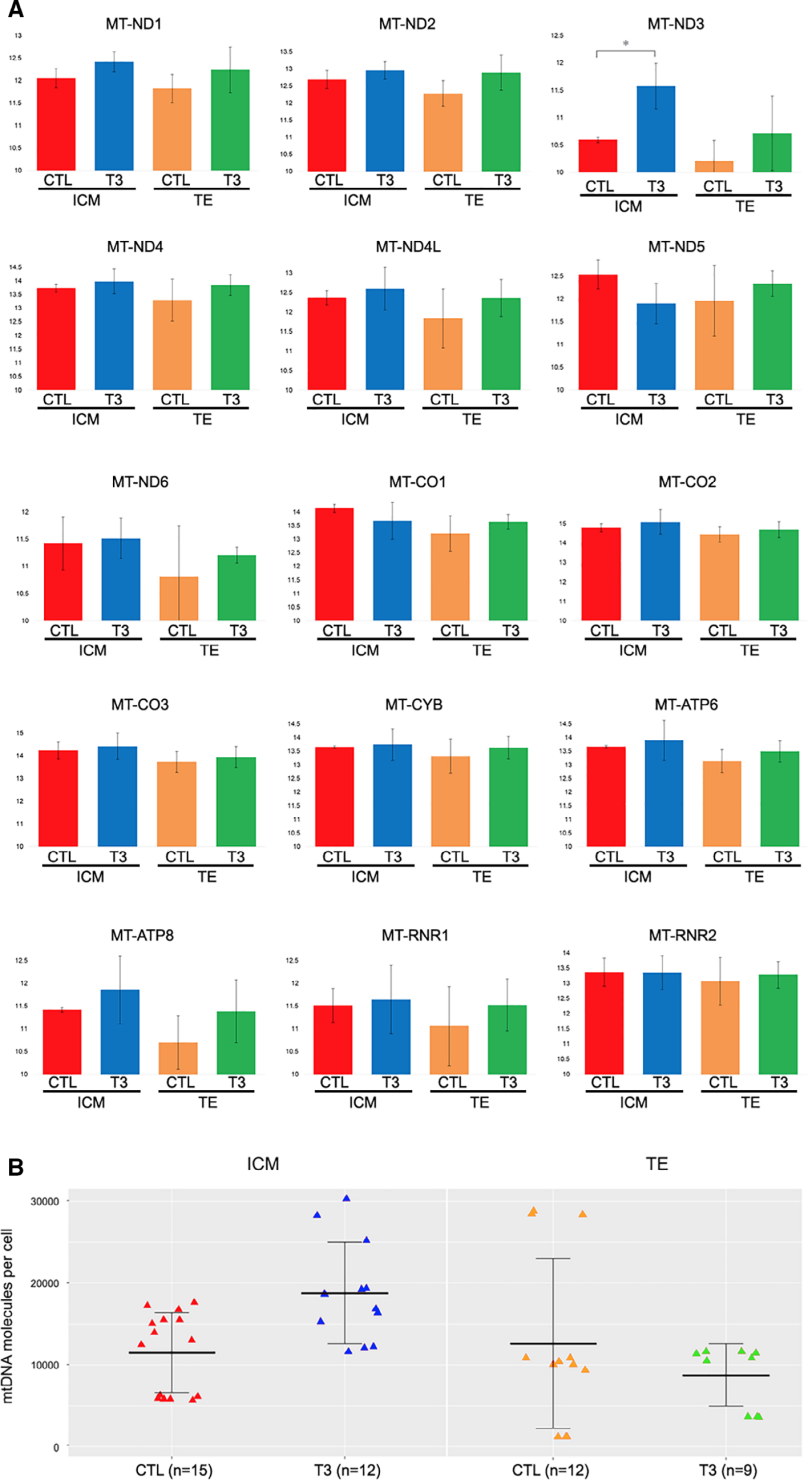
T3 effect on transcription of mtDNA encoded genes and mitochondrial biogenesis. A, Changes of individual mtDNA encoded genes. B, mtDNA content of single inner cell mass (ICM) and trophectoderm (TE) cells from five embryos in vivo. Each data point represents the mtDNA content in a single cell corresponding to the mean value from the independent qPCR measurements. Wilcoxon rank sum test has shown significant differences between the untreated control (CTL) and T3‐treated samples ICM cells (W = 143; *P* = .0087), whereas TE cells lacked the statistically significant difference between the control and T3‐treated groups (W = 60, *P* = .7021)

Mitochondrial DNA is circular in shape and contains 16 569 base pairs.^15^ The entire human mitochondrial genome encodes 37 genes, 22 of them code for transport RNA (tRNA), 2 code for ribosomal RNA (rRNA, *RNR1*, and *RNR2*), and 13 code for proteins that are necessary for cellular energy production: cytochrome b (*CYTB*), cytochrome oxidase 1, 2, and 3 (*COX*1, 2, and 3), ATP synthase 6 and 8 (*ATP*6 and 8), NADH dehydrogenase 1, 2, 3 4, 4L, 5, and 6 (*ND*1, 2, 3, 4,4L, 5, and 6; Figure 5A). Among these, we found that only *MT‐ND3* was significantly increased in cells of ICM exposed to T3. This suggests that, in the case of T3, the primary effect of T3 was on the nuclear, not the mitochondrial genome.

The T3‐induced shift in OXPHOS activity in ICM may not simply be a result of upregulation of the genes that are contributing to the composition and organization of the respiratory chain; the shift could also indicate an increase in the number of mitochondria.^16^ Indeed, mtDNA copy number as determined with qPCR was significantly increased in ICM and not in TE of T3‐treated embryos (Figure 5B), suggesting a crosstalk between the nuclear genome and mitochondrial replication. The nature of the crosstalk is currently unknown and remains to be investigated.

## 4 DISCUSSION

Thyroid hormones are major regulators of metabolic rate. Although the mechanism is not well understood, they have a profound effect on mitochondrial energetics, adenosine triphosphate (ATP) production, and OXPHOS. Studies on metabolism of bovine embryos have shown that ICM and TE have pronounced differences in carbohydrate metabolism, which may reflect their diverging roles in embryological development.^17^ The ICM had lower pyruvate consumption per cell than did the isolated TE, which is indicative of very low oxidative metabolism. Glycolytic index of TE indicated that all the glucose consumed was likely converted to lactate.

Transmission electron microscopic analyses of mitochondria in human preimplantation embryos have shown that dramatic changes in mitochondrial morphology occur during the differentiation of blastomeres in TE and ICM. Rounded to oval “immature” mitochondria are transformed into elongated tubular forms with well‐defined transverse cristae, a sign of increased metabolic activity.^18^

Mitochondria have been shown to be the major sites of T3 accumulation in cells, potentially having a direct effect on mitochondrial activity.^19^ Indeed, subsequent studies have demonstrated that multiple isoforms of THRA and THRB are present in mitochondria.^20^, ^21^, ^22^ T3 can affect mitochondrial function both directly, regulating expression from the mitochondrial genome through *THRA1* isoform p43 which may bind directly to the mitochondrial DNA,^20^ and indirectly, through the regulation of nuclear gene transcription of the mitochondrial transcription Factor A (*TFAM*)^23^ or regulators of mitochondrial biogenesis such as proliferator‐activated receptor delta (*PPARD*), nuclear respiratory factor‐1 (*NRF1*),^24^ mtDNA‐specific polymerase (*POLG*), or peroxisome‐proliferator‐activated receptor gamma coactivator‐1a (*PPARGC1A*).^25^

Although our data suggest that T3 induced mitochondrial biogenesis in ICM, we did not detect an increase in the expression of these genes, which suggests that our observed increase in number of mitochondria was mediated through a different pathway or pathways. For example, recently, a group of small non‐coding RNAs (sncRNA), known as P‐element–induced wimpy testis (piwi)‐interacting RNAs (piRNA), corresponding to mtDNA, have been described.^26^ Nuclear DNA‐encoded piwi‐like RNA‐mediated gene silencing 1 (PIWIL1) protein was detected in both nuclear and mitochondrial fractions suggesting a novel crosstalk between nuclear and mitochondrial genomes.^26^ mtDNA‐encoded piRNA was quite abundant in germ cells, gametes, and zygotes.^27^

Mitochondrial respiration contributes to energetic requirements during embryonic development. In the cleavage stage only 10% of available glucose is metabolized through aerobic respiration, whereas this is increased at the blastocyst stage to 85%.^28^, ^29^ Oxygen consumption has been linked to reproductive competence in mouse. Increase in oxygen consumption in cleavage stage embryos may indicate subsequent development to expanded blastocysts.^30^ Molecular oxygen is an essential component as it acts as the electron acceptor in OXPHOS. Maturation of mitochondria and transition from the glycolytic pathway to OXPHOS at the blastocyst stage is, therefore, likely to be linked to exposure to oxygen from maternal blood following embryo implantation. Although OXPHOS is more efficient in energy production and can generate up to 36 net molecules of ATP per one molecule of glucose, compared with to two net molecules of ATP per glucose in the glycolytic pathway, it generates reactive oxidative species; these are neutralized by the maintenance of redox balance by the NADPH/NADP+ ratio. This process may not be efficient during the earliest stages of embryo development,^31^ resulting in mitochondrial dysfunction, which has been proposed as a trigger for apoptosis in the embryo.^32^ Mitochondria may, therefore, represent a QC system that will determine whether the embryo proceeds further into development. Comprehensive genetic analyses, a combination of microarray comparative genomic hybridization, quantitative PCR, and next‐generation sequencing, provided information on chromosomal status, amount of mtDNA, and the presence of mutations in the mitochondrial genome and supported this hypothesis. The data suggested that successfully implanted blastocysts tended to contain lower mtDNA levels than those failing to implant.^33^ The study also suggested that the quantity of mtDNA was significantly higher in embryos from older women and in aneuploid embryos, unrelated to age.

In contrast, mitochondrial transplantation has recently emerged as a potential treatment of various diseases, including restoration of fertility.^34^ The first babies born following autologous mitochondria transfer from granular cells were born in 2003 in China.^35^, ^36^ Twelve years later, Woods and Tilly^37^ proposed that transplantation of healthy mitochondria into eggs from older women would improve the quality of the zygote and increase the rate of pregnancy.^38^

The association of reproductive competence with mitochondrial quantity and function at different stages of human embryo development remains unclear. However, the clinical evidence strongly suggests that T3 plays an indispensable role in the earliest stages of development. It is plausible to hypothesize that this is due to its effects on energy production and mitochondrial biogenesis, as found in the data presented here. Currently, the media used for culturing human embryos in vitro do not contain T3. We suggest that supplementing commercial media with physiological concentrations of T3 might have a beneficial effect on the embryo development and improve implantation and pregnancy rate.

Mitochondrial metabolism can also be considered as an alternative indicator of developmental identity and indicator of pluripotency stage. hESCs are considered to represent a later, postimplantation, phase of epiblast development, more similar to mouse epiblast stem cells.^39^, ^40^, ^41^ Based on transcriptional and protein expression profiles as well as on their epigenetic state, recently derived naive hESCs are more similar to mouse embryonic stem cells (mESCs) and ICM of preimplantation embryos.^42^, ^43^ The majority of mitochondria in mESC and naive hESC are rounded to oval, displaying sparse and irregular cristae and an electron‐lucent matrix.^42^, ^44^ Mitochondria in hESC and mEpiSC are elongated, contain well‐defined cristae and a dense matrix.^44^ Whereas both hESC and mEpiSC are dependent primarily on aerobic glycolysis, mESCs are bivalent and can convert between glycolytic and aglycolytic metabolism in an HIF1A‐dependent manner.^44^ However, despite having a more developed and expanding mitochondrial content, mEpiSCs/hESCs have low mitochondrial respiratory capacity due to low *COX* expression. T3 regulates the expression of COX subunits by both transcriptional and posttranscriptional mechanisms in a mitochondrial content‐dependent manner.^45^ Whether and how T3 is involved in any switch from ground stage of pluripotency of naive hESC to primed pluripotency of hESC remains to be determined.

## 5 CONCLUSION

Little is known about the effects of the thyroid hormones in the earliest stages of human development. However, women of fertile age with underactive thyroid glands are prone to suffer from menstrual irregularities and, although ovulation and conception are possible, resulting pregnancies are prone to miscarriage.

Our results indicate that thyroid hormones affect mitochondrial function in human embryos; although transcription of mitochondrial DNA (mtDNA) genes was not affected by T3, mitochondrial replication was stimulated, as was energy production within mitochondria by switching metabolism from the glycolytic pathway to more efficient OXPHOS. This effect was restricted to the ICM, the part of the embryo that gives rise to the body of the fetus.

Our report suggests a new mechanism of metabolic switching in human preimplantation embryos and highlights an important role of thyroid hormones in the early stages of pregnancy. It is plausible to hypothesize that supplementing commercial media with physiological concentrations of thyroid hormone might have a beneficial effect on embryo development and improve implantation and pregnancy rate.

Understanding of the basic biochemistry and roles of the ICM and TE in homeostasis of the blastocyst is still limited. One hypothesis is that the TE is a transporting epithelium, sparing nutrients for use by the ICM.^46^ More recent studies in mouse embryos have demonstrated that the TE consumes significantly more oxygen, produces more ATP, and contains a greater number and more mature mitochondria than the ICM.^47^, ^48^ The data from our study, although limited, suggests that such a balance in energy production between pluripotent ICM and differentiated TE cells might also be present in human embryos. The role of T3‐induced metabolic changes in differentiation of ICM cells is likely to be a fruitful area for future studies.

## CONFLICT OF INTEREST

The authors indicated no potential conflicts of interest.

## AUTHOR CONTRIBUTIONS

L.N., S.E.K.: conception and design, collection and/or assembly of data, data analysis and interpretation, manuscript writing, final approval of manuscript; A.P.: collection and/or assembly of data, data analysis and interpretation; G.G., N.F., A. Capalbo, L.D., P.K., H.R., N.K., K.K.N.: collection and/or assembly of data; V.M.J., A.M., N.H: data analysis and interpretation; A. Cvoro: conception and design, data analysis and interpretation, manuscript writing; R.F.: provision of study material or patients; M.S.A.: financial support, final approval of manuscript; P.F.C.: conception and design, collection and/or assembly of data, data analysis and interpretation, final approval of manuscript; C.O.: conception and design, data analysis and interpretation, manuscript writing, final approval of manuscript; Y.K., D.I.: conception and design, data analysis and interpretation, manuscript writing, financial support, final approval of manuscript.

## Supporting information


**Figure S1** Expression pattern of lineage specific markers indicate separation of two subpopulations.
(**A**) Expression of ICM lineage markers *NANOG*, *NODAL*, and *SOX2*.
(**B**) Expression of TE lineage markers *CDX2*, *GATA2*, and *GATA3*.

**Figure S2** In human preimplantation embryos, both thyroid hormone receptors, *THRA* and *THRB*, are expressed predominantly in ICM.

**Table S1** List of all transcripts detected in this study, with their Reads Per Kilobase of transcript per Million mapped reads (RPKM) values. As indicated in Methods, differential analyses were made using Deseq2 from normalized gene counts, not from RPKM. All normalized gene counts used for this analysis are accessible on GEO under accession number GSE131499.

## Data Availability

The data have been deposited in the Gene Expression Omnibus (GEO) under accession number GSE131499.
